# Standard approach and future perspective for the management of benign prostatic hyperplasia from a health-economics point of view: the role of transperineal laser ablation

**DOI:** 10.3389/fruro.2023.1100386

**Published:** 2023-02-24

**Authors:** Valentina Lorenzoni, Ilaria Palla, Guglielmo Manenti, Pasquale Ditonno, Theo M. de Reijke, Giuseppe Turchetti

**Affiliations:** ^1^ Institute of Management, Scuola Superiore Sant’Anna, Pisa, Italy; ^2^ Department of Diagnostic Imaging and Interventional Radiology, PTV, Rome, Italy; ^3^ Urology, Policlinico Università di Bari, Bari, Italy; ^4^ Andros Clinics, Amsterdam & Department of Urology, Amsterdam University Medical Centers, University of Amsterdam, Amsterdam, Netherlands

**Keywords:** benign prostatic hyperplasia (BPH), quality of life, innovative, lower urinary tract symptoms, TPLA, EchoLaser, Cost analysis

## Abstract

**Introduction:**

Benign prostatic hyperplasia (BPH) is a common diagnosis among the ageing male population over 60 years and it is associated with the development of lower urinary tract symptoms (LUTS): dysuria, nocturia, increased frequency of urination, etc. LUTS negatively affect the patient’s daily activities and the quality of life. Patients with severe and persisting symptoms, not responding to pharmacological therapy, are candidates for surgical intervention. Transurethral resection of the prostate (TURP) has been the gold standard for surgical approach despite it can be associated with significant complications. Indeed, laser vaporization or enucleation are today the most broadly used surgical techniques and other minimally invasive surgical therapies (MISTs) have been introduced to reduce some complications during- and post-surgery. Moreover, a new micro-invasive approach for LUTS is represented by EchoLaser SoracteLite™ transperineal laser ablation (TPLA), an innovative, safe and feasible approach that can be performed under local anaesthesia and in an outpatient setting.

**Objective:**

The paper aims to analyse and discuss the economic implications of standard surgical techniques and innovative approaches with a focus on TPLA thought a literature review.

**Results:**

The literature review highlights that at present there are few studies related to the economic implications of surgical therapies for LUTS. Preliminary results show that the TPLA is a promising technique in terms of clinical and economic benefit for the treatment of obstructive LUTS. Furthermore, TPLA can be performed in an outpatient setting implying an advantage from an economic and also organizational point of view, in particular in a health emergency situation.

**Conclusions:**

Economic literature on minimally invasive techniques and surgical approaches for the treatment of BPH is still lacking. Multicentre and long-term economic studies are needed to assess the estimated disease burden. However, direct and indirect costs associated with TPLA are minimized vs TURP and laser vaporization/enucleation.

## Introduction

Benign prostatic hyperplasia (BPH) is a non-cancerous enlargement of the prostate affecting more than 50% of men above 60 years (1). BPH is linked to the development of lower urinary tract symptoms (LUTS): dysuria, nocturia, increased frequency of urination etc. The obstructive symptoms can lead to complications such as urinary retention, urinary tract infection, haematuria and hydronephrosis. LUTS negatively affect the patient’s daily activities and Quality of Life (QoL), also including psychological stress due to anxiety (2). LUTS are strongly associated with ageing and several modifiable risk factors (3, 4). Therefore, prevalence of the disease and associated costs are likely to increase with future demographic changes (5).

Treatment of BPH-related LUTS depends on the severity of the problem that is usually defined based on the International Prostate Symptom Score (IPSS) which distinguishes mild (score from 0 to 7), moderate (score from 8 to 19) and severe symptoms (scores from 20 to 35) (6).

Common clinical practice foresees initial treatment of patients with moderate to severe symptoms with pharmacotherapy (α-blocker, 5α-reductase inhibitor or combined therapy). Low compliance, intolerance or allergy, and costs to maintain therapy are major disadvantages that have a negative impact on effectiveness of this approach. Moreover, there is recent evidence that medical therapy for LUTS due to BPH increases the risk of cardiac failure and stroke (7), the risk of suicide and psychological adverse events (8) and dementia (9).

Patients with severe and persisting symptoms, not responding to pharmaceutical therapy, are candidates for surgical intervention. Among these, transurethral resection of the prostate (TURP) has long been considered the gold standard; it can however be associated with significant complications such as bleeding, urinary incontinence and retrograde ejaculation (10) and it is performed under regional or general anaesthesia which requires the patient be treated in inpatient setting. Laser therapy such as Holmium Laser Enucleation of the prostate (HoLEP), Thulium and Greenlight laser therapy are implemented with the aim to reduce perioperative bleeding (and post-op blood transfusions) and are emerging as a new standard for BPH surgery (11).

Other minimally invasive surgical therapies (MISTs) have been introduced in the past years being nowadays available and recognized (although as “investigational”) also by the American Urology Association (AUA) and the European Association of Urology (EAU) (11). In detail, thermo-ablative strategies such as transurethral microwave therapy (TUMT) and transurethral vaporization of the prostate (TUVP), mechanical therapy as Urolift and intra-prostatic stent (iTIND), water based treatment as Aquablation, Rezūm system, an ablative system, prostatic artery embolization (PAE) and the use of Intraprostatic Injectables could be considered depending on the availability of the procedure, patient risk, prostate volume, patients’ preference and the sustainability of the approaches. New techniques such as Aquablation use a transurethral approach implying similar complications as TURP; furthermore, Aquablation needs general anaesthesia. Rezūm, which is a thermal therapy based on a transurethral approach, leads to high risk of urethral damage with irritative symptoms in the post-operative period and haematuria. A large-scale analysis of real-world healthcare data for enlarged prostate procedures presented at the American Urological Association 2021 Annual Meeting, revealed that surgical re-treatment rates are comparable among the UroLift System, TURP and GreenLight, while highest for Rezūm (12).

EchoLaser SoracteLite™ transperineal laser ablation (TPLA) represents an innovative, safe and feasible micro-invasive approach for LUTS treatment (13, 14) that can be performed under local anaesthesia in an outpatient setting using thin introducer needles (21G). Furthermore, a recent study (15) shows that the TPLA ensures good and stable results after three years from the treatment.

The present paper aims to review and discuss the economic implications of standard surgical techniques and innovative approaches with a focus on TPLA.

## Recommended surgical approaches

TURP has been considered the gold standard for the management of LUTS secondary to benign prostatic hyperplasia. In the last years, several techniques have been developed as safe and effective alternatives [2022 EAU Guidelines].

Despite invasiveness of the procedure that involves the insertion of a resectoscope through the urethra to remove obstructing tissue, surgical and follow-up complications have been shown to be reduced over years with increasing experience. Moreover, alternatives have been developed and made available for patients depending on the prostate volumes even if long-term effects on relapses and complications of those alternatives are still lacking (16).

Endoscopic enucleation of the prostate (EEP) is a laser-based (Holmium, Thulium, Greenlight-PVP) and non-laser-based (monopolar, bipolar, and plasmakinetic) transurethral approach, able to enucleate completely the prostatic adenoma similar to an open prostatectomy without the side effects of an open surgery. HoLEP is emerging as a new gold standard for the surgical management of the high-volume BPH, but it is a difficult technique with a high learning curve (15). Thulium laser therapy is similar in terms of complications and re-interventions after surgery compared to HoLEP (17).

Prostate vaporization is achieved by heating the prostatic adenoma trough high-energy application. This can be accomplished by either a bipolar electrical system (TUVP) or laser energy with Greenlight. These procedures demonstrated the safety and efficacy for treatment of prostates up to 70 grams while the durability of the outcome remains a concern.

Short-term studies showed efficacy and safety of the above mentioned procedures that could be comparable with TURP, with generally a reduced hospital length of stay (18).

Main characteristics of the available approaches are detailed in **Table 1**.

**Table 1:** The main approaches to manage LUTS due to benign prostatic hyperplasia.

Approaches	Description
Surgical therapy	
Transurethral resection of prostate (TURP)	TURP removes tissue from the transition zone of the gland.
Transurethral Incision of the Prostate (TUIP)	TUIP involves incising the bladder outlet without tissue removal.
Transurethral vaporization of the prostate (TUVP)	TUVP is a modification of existing transurethral technology: an instrument is inserted through the urethra into the prostate. A ball or special wire loop on the instrument heats the prostate tissue and turns it into vapor.
Prostatic artery embolization (PAE)	Prostatic Artery Embolization can be performed in an outpatient setting under local anaesthesia with access through the femoral or radial arteries.
Mechanical therapy	
Prostatic urethral lift (PUL)	PUL is a novel minimally invasive approach performed under local or general anaesthesia. Encroaching lateral lobes are compressed by small permanent suture-based implants delivered under cystoscopic guidance (Urolift^®^).
Intraprostatic stent (iTIND)	The iTIND is a device designed to remodel the bladder neck and the prostatic urethra. This is left in position for five days and it is removed by standard urethroscopy in an outpatient setting.
Water based treatment	
Aquablation - image guided robotic waterjet ablation	Aquablation is a new technology that utilizes machine-controlled water jets to ablate the soft tissue of the prostate.
Rezūm system: convective water vapour energy (WAVE) ablation	The Rezūm system uses radiofrequency power to create thermal energy in the form of water vapor, which in turn deposits the stored thermal energy when the steam phase shifts to the liquid phase upon cell contact. The steam disperses through the tissue interstices and releases stored thermal energy onto prostatic tissue effecting cell necrosis. The procedure can be performed in an office-based setting. Usually, one to three injections are needed for each lateral lobe and one to two injections may be delivered into the median lobe.
Laser therapy	
Holmium Laser enucleation of the prostate (HoLEP)	A pulsed solid-state laser that is absorbed by water and water-containing tissues and in this way the adenoma can be removed.
Thulium laser therapy	Thulium laser and pulsed Holmium laser offer complete absorption of laser energy in water. Furthermore, the Thulium laser offers advanced vaporization and haemostatic features.
Greenlight laser therapy	The technique uses a laser to rapidly heat and vaporize the excess prostate tissue; removing the excess tissue rapidly restores natural urine flow in most patients. The GreenLight laser procedure is typically performed in an outpatient and inpatient setting under general or epidural anaesthesia.

## Novel perspective among surgical approaches

**Figure 1:**
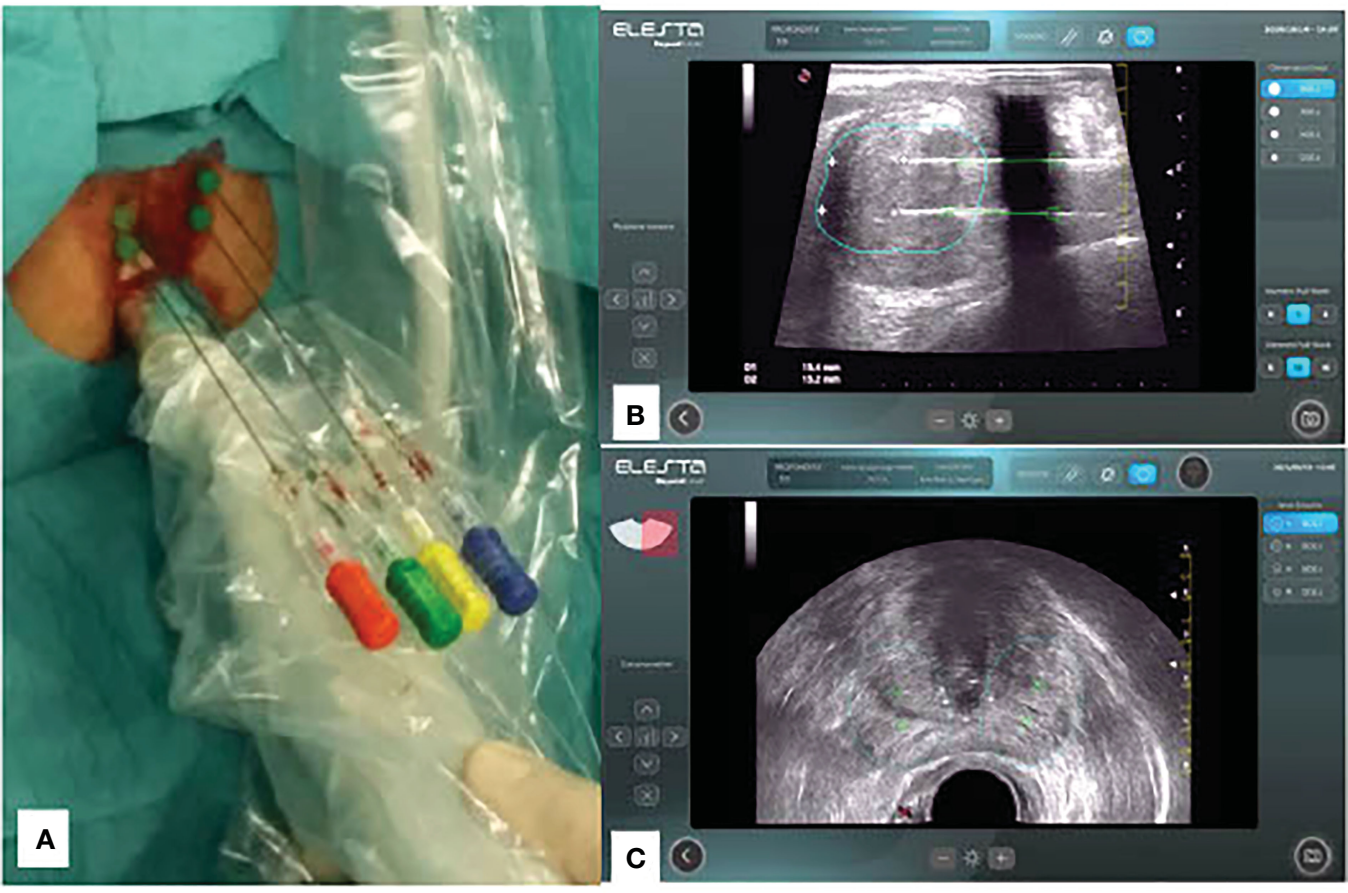
**(A)** Transperineal approach with 4 fibers placed in position and monitored by US images coming from Biplane probe; **(B)** Treatment Planning: longitudinal plane with two fibers positioned at fair safety distances from prostate capsule (@1800J) including 1 Pull-Back of 10mm; **(C)** Treatment Planning: transverse plane with planning of needle/fiber position according safety distances at 1800J.

Patelli et al. (14) presented the first data on TransPerineal Laser Ablation with a good efficacy and safety profile that should be confirmed in larger studies. For a TPLA procedure the patient is positioned in the lithotomy position and a three-way 18-F Foley catheter is inserted to permit cooling irrigation during the whole lasing period in order to prevent injuries to the urethral wall. Some users prefer to place normal 2 way catheter. Following local anaesthesia of the perineal region and periprostatic plexus, the procedure is carried out under transrectal ultrasound (US) guidance. One or two 21 G introducer needles (EchoLaser introducer needle, Elesta SpA, Calenzano) for each lobe are simultaneously inserted transperineally in the adenoma and placed as parallel as possible in the longitudinal plane of the prostate (**Figure 1A**). In order to facilitate the insertion of the needles, the transrectal US biplanar probe is combined with a multi-channel needle guide, with a dedicated planning tool device (Echolaser Smart Interface, Elesta SpA, Calenzano) with a software displaying needle trajectories and safety area overlying the US image in the longitudinal plane (**Figure 1B**) and transverse plane (**Figure 1C**).

**Figure 2:**
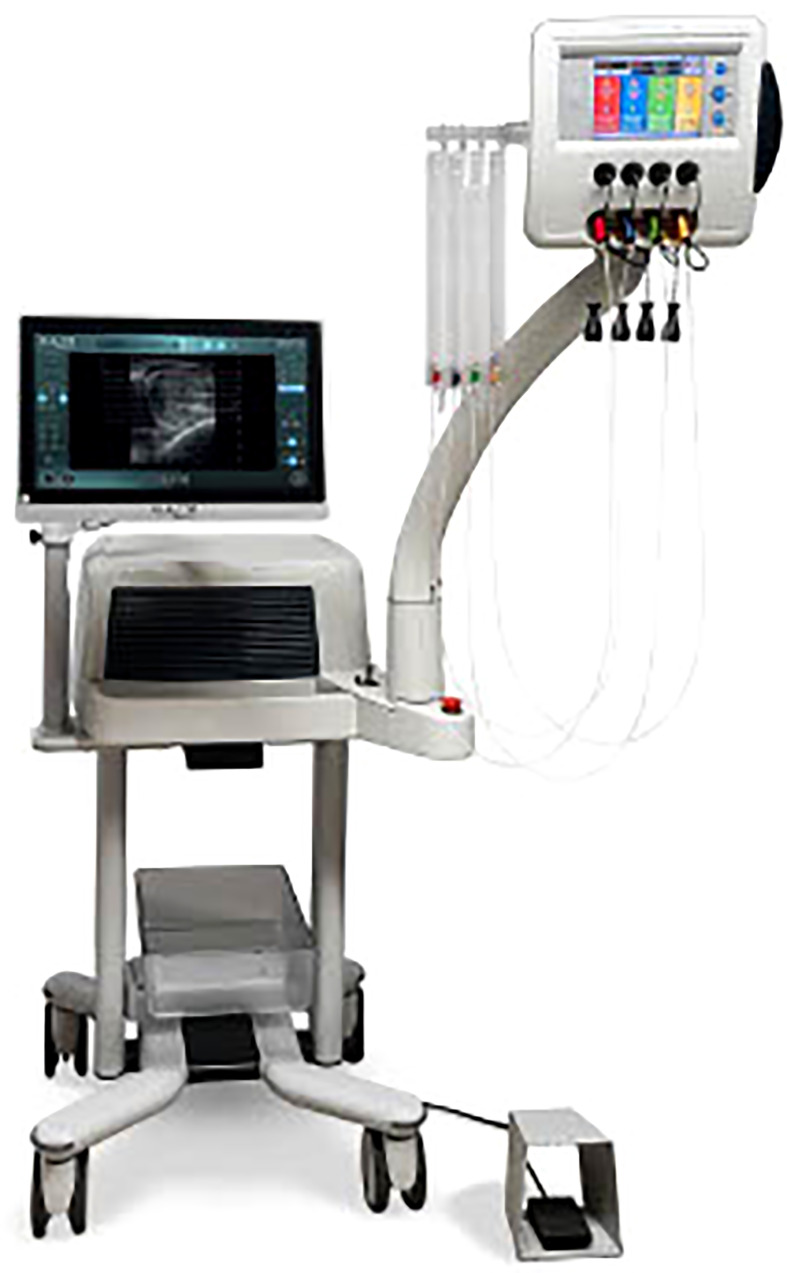
EchoLaser consists of a multisource laser and a touch panel for treatment planning (Echolaser Smart Interface).

Subsequently, one 272µm flat-tip optical fiber (EchoLaser Fiber Optic for PLA, Elesta SpA, Calenzano) per needle is introduced. The safety area is determined after introduction of the laser fibers. The optical fibers are then connected with a continuous wave diode laser source (EchoLaser, Elesta SpA, Calenzano) (**Figure 2**) and energy delivery is performed with a fixed power (3W, 1800 J - however it has been observed by some users that optimal outcome might be achieved with higher power, up to 5W, which in their experience was shown to be well tolerated by non-anaesthetized patients) (19).The number of applicators per prostatic lobe and the number of pull-back are chosen according to prostate shape and volume taking into account safety distance from urethra, prostatic capsule and bladder neck. The use of Echolaser Smart Interface device for the treatment planning helps in positioning the optical fibers through a graphical representation of a safety area even in the case of multiple applicators. Correct positioning of the fiber tips preserve the anatomical structures such as bladder neck, verumontanum, urethra and sphincter and thus preserving antegrade ejaculation and urinary continence (19) and reducing post-op complications and side effects.

Several retrospective and prospective series with intermediate term of follow-up demonstrated that TPLA is a safe and feasible procedure for the treatment of LUTS due to BPH, with a good safety profile. Cai et al. (20) and Pacella et al. (21) retrospectively and Frego et al. (22), De Rienzo et al. (23) prospectively demonstrated a significant improvement in IPSS score, in peak urinary flow rate (Qmax), post void residual, a reduction in prostate volume at 3 months and improvement in QoL at follow-up. The study performed by Cai et al. (20) shows that the IPSS score improved from 22.7 ± 5.3 (baseline) to 9.1± 3.2 (after 6 months); the Qmax improved from 8.5 ± 3.0 to 15.2 ± 4.8 mL/s (P < 0.001), the PVR increased from 78.7± 58.8 to 30.3 ± 34.2 (P<0.05), and the mean prostate volume ranged from 70.8 ± 23.8 to 54.7± 20.9 mL (P<0.05). In the study of Pacella et al. (21), at 12 months the mean IPSS score improved from 22.5 ± 4.5 to 7.0 ± 2.9 (P < 0.001); the PVR from 71.7 ± 93.9 to 17.8 ± 51.0 ml (P < 0.001), Qmax from 8.6 ± 5.2 to 15.0 ± 4.0 ml/s (P < 0.001) and the QoL from 4.2 ± 0.6 to 1.6 ± 0.9 (P < 0.001).

In Frego et al. (22), IPSS score decreases from 22 at 6 points; the median postoperative Qmax improved by +57.8%, +98%, and +115.8%, at 3, 6, and 12 months; the post void residual decreases from 60 ml a 30 ml; the median prostate volume significantly decreased by a − 21.3%, − 29%, and − 41%, respectively at 3, 6 and 12 months. In De Rienzo et al. (23), the IPSS score amounts to 18.3 ± 3.9 in the preoperative phase and to 6.1 ± 2.6 at 6 months; the Qmax improved from 9.2 ± 3.4 (preoperative) to 13.9 ± 6.2 (6 months); the post void residual decreases from 81.8 ± 62.6 (preoperative) to 14.0 ± 16.7 (6 months). Furthermore, the ejaculatory function is maintained at all follow up visits up to 12 months (21) and the sexual function is preserved in all sexually active patients (19).

The study performed by Manenti et al. (24) assessing the impact of ultrasound-guided TPLA shows that the procedure is a safe, manageable and effective treatment for LUTS. The patients had an improvement in urinary symptoms, preserve the sexual and erectile function. The results at 12 months in terms of IPSS score, PVR, Qmax and QoL are comparable with the other cited studies in the paper.

The recent study performed by Sessa et al. (25) aimed to assess the early functional and sexual outcomes in 38 patients underwent to TPLA between April and February 2022 in a single center. The study showed that the median time of procedure was 31 minutes (IQR 28-37) and 37/38 patients were discharged within 8 hours of hospital stay. Qmax improved from 9.1 (8.0-11.5) at baseline to 10.6 (9.0-13.6) at 1 month, 11 (9.4-13.6) at 12 (9.5-15.0) at last follow up (range 4-12 months). The median postoperative IPSS decreased by -14%, -36% and -35% at 1 month, 3 months and at last follow up. Ejaculation was preserved in all patients.

From the study perfomed by Gerbasi et al. (15) emerged that 20/21 patients submitted to TPLA from September 2018 to March 2019 presented a significant improvement in IPSS (-37.2%) and QMax (+45.8%) after three years of the treatment. The authors underlined that the ultra-minimally invasive surgical approach could be an alternative to medical therapy and more invasive surgical approaches. Furthermore, from the study emerged that the improvement of the functional outcomes is maintained stable over the time after 3 years^1^.

In January 2023, Sessa et al. (26) have published a review with the aim to revise the current evidence on surgical and functional outcomes related to TPLA for LUTS due to BPH. The review included 7 studies of which 6 we have presented. The review empathizes the promising results in terms of functional outcomes and patient safety; but highlights the need to implement prospective studies to compare the results of TPLA versus other techniques. Recently, it was published the protocol of a randomized clinical trial comparing TPLA versus TURP aims to analyze the impact in terms of relief in benign prostatic obstruction and preservation of ejaculatory function at 1, 3 and 6 months (27).

However, randomized clinical trials in multicenter settings are desirable to demonstrate the advantages over a long period (22). Currently, it is published the study protocol of the first multicenter randomised clinical trial including 16 participating centres in China, Italy, Switzerland, and Poland aims to compare the efficacy and safety of TPLA respect to TURP (28).

## Health economics implications

With respect to the health economic implications, literature data showed that pharmacological treatment is less costly with respect to surgical treatment, but those approaches were also shown to be not cost-effective (16). To provide an overview about currently available non-pharmacological treatments, a literature review was performed as part of the present study. A review of the English language literature was perfomed using two databases (PubMed and Scopus) from 2011 to 2021 (**Additional File 1** for details about the methods and searching strategy used). An additional research was performed in February 2023 to research the latest evidence.

**Table 2** summarizes the results of 10 studies included in the literature review. Six of them performed a formal health economic evaluation, either a cost-utility (CUA) or a cost-effectiveness analysis (CEA) (29–31, 33, 34); 4 were simple cost-analyses (32, 35–37) and just one study also reported a budget impact analysis (BIA) (30). Almost all the studies performed a comparison just between Greenlight PVP (Photoselective Vaporization of the Prostate) and TURP (29–32, 34, 36). Ulchaker et al. (33) considered different available treatments including medical treatment, convective Radiofrequency (RF), prostatic urethral lift (UroLift^®^), PVP and TURP; Brown et al. (38) considered PAE, PVP and TURP and Ahn et al. (35) compared medical treatment versus surgery. Finally, Noble et al. (38) compared TURP and ThuVARP (Thulium laser transurethral vaporesection). Time period considered in the studies varied from 1-month to 2-years. In almost all studies only direct health costs were considered.

**Table 2:** Main characteristics and results of the study retrieved with the literature review.

Author, year	Country	Type of study	Perspective	Type of economic evaluation	Alternatives	Population	Costs	Results	Conclusion
Whelan et al., 2013 (29)	Canada	Prospective non randomized multicentre trial	Payer, 2011 Canadian dollars (CAD)	CUA over 6 months	TURP vs Greenlight PVP (HPS-120)	Patients diagnosed with symptomatic/ obstructive symptoms secondary to BPH requiring surgical intervention (ie., TURP), allocated to TURP, 24 patients, or PVP, 140 patients	Direct costs related to surgical intervention and follow-up visits, procedures and hospitalizations	TURP: $4,863 PVP: $3,771 (p-value=0.001) *QALYs* TURP: 0.441 PVP: 0.448 (p-value=0.658). PVP was less costly and more effective and had probability greater than 0.99 of being cost-effective for willingness to pay of $50 000/ QALY or $100 000/QALY	Despite PVP increased operating time, the outcomes are similar but PVP is a bloodless and painless procedure and could be performed outpatient requiring hospital admission for a small portion of patients.
Bowen et al., 2013 (30)	Canada	Prospective non multicentre randomized trial	Ministry of Health and Long-Term Care and societal perspectives, Canadian dollars	CEA over 6 months	TURP vs Greenlight PVP	BPH patients undergoing TURP 24 patients, or PVP 140	Direct and indirect costs related to surgical intervention and 6 and 24-month follow-up	Six months men cost per patient were significantly lower for PVP, CA$3,891 vs TURP (CA$4,863). Similarly for 24-month costs that were CA$4116 and CA$4946 respectively. 6-month QALYs were 0.448 for PVP and 0.441 for TURP, accordingly PVP was the most cost-effective option for different WTP thresholds. At BIA PVP was shown to induce savings over a 5-years period	PVP appears to be a cost-effective alternative to TURP, providing similar clinical benefit at a lower cost to the health system. Moreover the opportunity to avert inpatient stays and redirect funds to other areas by using PVP over TURP could free up over 28,000 inpatient days and just over CA$14 million for other uses.
Benejam-Gual et al., 2014 (31)	Spain	Retrospective study (not randomized)	Payer, Euro 2012	CUA, over 2 years	TURP vs Greenlight PVP (performed inpatient)	Patients presenting LUTS secondary to BPH undergoing TURP, 50 or PVP, 48 patients	Direct costs related to surgical intervention and 2-years follow-up	TURP: €3,770 PVP: € 3,377 *QALY* TURP: 1.636 PVP: 1.711 PVP was dominant implying significantly lower costs (-393€) and higher QALY (0.075)	PVP dominated TURP due to slightly higher effectiveness and lower costs. Overall costs were mainly driven by surgical costs while follow-up costs were the main responsible for differences between groups.
Hsu et al., 2016 (32)	Taiwan	Prospective study	Payer, 2005 New Taiwan dollars (NT$)	CA	TURP vs Greenlight PVP 120W	Patients with LUTS and BPH TURP: 100 PVP: 100	Direct costs, related to the intervention	Overall mean costs were 40,427 NT$ for TURP and 164,364 NT$ For PVP	PVP results more expensive compared to TURP and the difference was mainly due to significantly higher costs for equipment and accessories.
Ulchaker et al., 2018 (33)	USA	Model based study relying on data from literature and other sources	Payer, 2016 US dollars	CEA over 2 years	-Therapy with combination prescription drugs (an inhibitor 5∝-reductase + an ∝-selective adrenergic receptor blocker) **-**Radiofrequency (RF) thermal therapy procedure (Rezūm® System and Prostiva® RF therapy System); prostatic urethral lift (UroLift®) **Invasive procedure:** Greenlight PVP; TURP	Hypothetical cohort of patients with LUST/BPH and IPSS of 22	Direct health costs associated with the different BPH therapies	Rezum® had intermediate costs (lower than TURP and PVP) and intermediate effectiveness (lower than TUTP and PVP but higher than ComboRx) and TURP the most effective and costly approach. Among non-dominated therapies, ComboRx is the least expensive $1,736 and less effective, RF Prostiva® had intermediate costs ($2,855) and effectiveness higher than ComboRx, UroLift costs we $6,386 and it was dominated by Greenlight PVP which had lower costs and higher IPSS.	Medication therapy is least expensive but not cost effective. Among minimally invasive therapy only Rezum® has the potential to be cost-effective if 2-year benefit last longer despite it was less effective than PVP and TURP.
Erman et al., 2018 (34)	Canada	Microsimulation decision-analytic model	Public	CEA	TURP or GL-PVP as initial treatment versus pharmacotherapy as initial treatment followed by BPH surgery if symptoms do not solve. 8 strategies: upfront GL-PVP 5-ARI followed by delayed GL-PVP α-blocker followed by delayed GL-PVP combined therapy (5-ARI+α-blocker) followed by delayed GP-PVP upfront TURP 5-ARI followed by delayed TURP α-blocker followed by delayed TURP combined therapy (5-ARI+α-blocker) followed by delayed TURP	NA	Direct costs	Discounted lifetime costs ($CAN) 9,806.89 9,993.82 10,258, 43 10, 372.53 10,507.91 10,737.39 11,958.65 12,973.35 Upfront TURP is most costly and effective compared to upfront GL-PVP Upfront TURP costs $1015 more with a gain of 0.03 QALYs	Surgical approach results cost-effective.
Ahn et al., 2018 (35)	South Korea	Retrospective (hospital billing data)	NA	CA	Long term curative medical therapy (any medical therapy including a 5α-reductase inhibitor during ≥5 consecutive years or ≥1 year until surgery due to medical therapy failure) versus early surgery at <1 year of initial visit.	Medical therapy: 137 Early surgery: 70	Direct costs and out-of-pocket costs	*Mean direct costs* (inpatient+ outpatient) Medical therapy: $3987 Early surgery: $3036 *Out of pocket costs* Medical therapy: $1742 Early surgery:$1436	At the beginning of the pathway, the patient should know that the out of pocket cost at 5 years of continuous medication will exceed that of the early surgery costs.
Masucci et al., 2018 (36)	Canada	Retrospective chart review	Hospital, 2015 Canadian dollars	CA	Greenlight PVP vs TURP and bipolar TURP (resection using bipolar electrocautery)	BPH patients undergoing surgery from 2013-2015 at the Toronto Western Hospital: 56 treated with Greenlight PVP, 29 undergoing Bipolar TURP and 118 undergoing TURP	Direct health costs related to the procedure and 30 and 60-day readmission	Mean total cost per patient were lower for Greenlight PVP, CA$3836 and slightly higher for both Bipolar TURP and TURP, CA$4978 and CA$4963 respectively. The difference was confirmed when also adjusting for patients’ differences	Greenlight PVP allow decreasing per-patient costs being mainly performed as day surgery (on the opposite of TURP and bipolar TURP) and decreasing readmission rates
Brown et al., 2019 (37)	Canada	Retrospective chart review	Hospital, 2017 US dollars	CA	TURP, PVP, and PAE	BPH patients undergoing TURP, 209 patients PVP, 29 patients, PAE, 21 patients, from 2015 to 2017	Direct health costs related to the procedure and 30-day readmission	Mean total per-patient costs were significantly lower for PAE, $3821, followed TURP, $5034, and PVP, $5719	PAE has fewer complications and shorten length of stay (1 day versus 1.55 and 1.63, PVP and TURP respectively), that implies a lower per-patient costs associated to PAE irrespectively of higher procedural costs
Noble et al., 2020 (38)	UK	Multicentre, pragmatic, randomised, controlled, parallel group trial	Healthcare, 2016/17 pounds	CEA over 12 months	TURP and ThuVARP	410 BPH patients agreeing to participate into the study and randomised 1:1	Direct health costs	From the NHS perspective, mean total costs were similar between TURP, £4305, And ThuVARP, £4309. Similarly, for QALYs that were 0.4 and 0.83 for TURP and ThuVARP respectively	The greater length of time needed to conduct the ThuVARP procedure meant the ThuVARP arm remained slightly more expensive, despite longer recovery time and slightly greater postoperative resource use for patients in the TURP arm, the probability TURP is cost-effective for a WTP of at least £20000/QALY was good.

CUA, Cost-Utility Analysis; CEA, Cost-Effectiveness Analysis; CA, Cost Analysis; TUVP, transurethral vaporization of the prostate; HoLEP, holmium laser enucleation of the prostate; PAE, PVP, photoselective vaporization of the prostate; ThuVARP, thulium laser transurethral resection; TUNA, transurethral needle ablation; TUMT, transurethral microwave thermotherapy; PUL, prostatic urethral lift; KTP, potassium titanyl phosphate (80 W); LBO, lithium borate (120 W); GL-XPS, green light XPS laser system (180 W); WTP, willingness to pay threshold.

Despite TURP is considered the gold standard according to a clinical perspective, novel alternatives such as PVP (GreenLight laser) were shown to be cost-effective compared to TURP in almost all studies considering those alternatives (27, 31, 33, 34). In detail, PVP has been shown to be performed mainly as an outpatient procedure or day-surgery thus reducing costs associated with hospital stay, but also imply lower readmissions and post-operative complications (ie, bleeding) possibly. The study comparing PAE, PVP and TURP showed that PAE was less costly compared to TURP and PVP; PAE has fewer complications and a shortened length of hospital stay (37). ThuVARP has been shown to be not a cost-effective alternative to TURP (**Table 2**) (38).

Studies related to the economic impact of TPLA as a micro-invasive technique are scarce.

One abstract presented at the European Congress of Radiology in 2020 provided a preliminary evaluation of the economic implications of the TPLA (39).

An assessment was performed according to several therapeutic options available for the treatment of LUTS due to BPH. They were identified in: Open Surgery, TURP, HoLEP and TPLA.

The therapeutic process is divided into two macro-phases, each of which has been coded as follows:

- Treatment activities (TA);
- Post-treatment activities (PTA);

In detail the Treatment Activities include: preparation of the room and of the patient and the treatment. Post-treatment activities include mainly costs of post-operative hospitalizations (**Table 3**).

**Table 3:** Operative costs of different therapeutic options for BPH (€).

Assessment		Cost per unit(euro)	OP	OPCost	TURP	TURPCosts	HoLEP	HoLEPCosts	PVP	PVPCosts	TPLA	CostTPLA
**Treatment Activities**	Surgery Room (min)	12	45	540	53	636	72	864	90	1080	0	0
	Medical Personnel	53,17	4,5	239	2,5	133	1,2	64	1	53	1	53,17
	Nurse assistance	19,64	8	958	4	369	2	110	2	86,5	1	43
	Materials		200	200	250	250	150	150	1300	1300	3	1800
	Hemotransfusion	151	0,75	113	0,4	60,4	0,05	7,55	0	0	0	0
	Pharmacotherapy			244		188		112		88		0
**Post-operative activities (days)**		674	6,1	4111	4,7	3167	2,8	1887	2,2	1482	0	
**Lease of equipment**											1	600
***Total (Euro)***				***6406***		***4804***		***3194***		***4090***		***2496***

OP, open prostatectomy.

TPLA costs were compared to those of open prostatectomy (OP), TURP, HoLEP and PVP showing that TPLA has lower perioperative costs with respect to the other techniques (TPLA: €2496, OP: €6406, TURP: €4804, HoLEP: €3194 and PVP: €4090).

Despite the paucity of economic evidence available about TPLA, given the characteristics of the technology and the available preliminary clinical data, some potential economic implications related to the use of the technology could be delineated. On one hand, the acquisition costs of the technology may weigh on the costs of intervention as compared to less costly options such as TURP. However, in the above mentioned abstract a lease cost of 600€ for the capital equipment was factored in, along with an average use of three consumable fiber kits per procedure (at 600€ each, for a total of 1800€). On the other hand, the possibility to perform the procedure in an outpatient setting, the micro-invasive nature of the intervention and the claimed benefit on QoL and complications, if confirmed in future studies, may reduce both direct health costs in the postoperative phase and during follow-up but also direct non-health and indirect costs (20–23).

## Discussion

The present study provides an overview of the economic and health economic implications of procedures for LUTS secondary to BPH.

First of all, the literature review highlights that at present there is not an abundance of studies related to the economic implications of surgical therapies for LUTS. Moreover, most of the evidence available is related to the comparison of TURP versus PVP but recently some studies explore the economic dimensions of MISTs. Recently, a cost-minimization and budget impact analysis showed that over 4 years the costs per patient with Rezūm were €2072 versus €2836 withTURP and the Rezūm is highly to be cost-saving approach compared with TURP from an Italian hospital healthcare perspective (40). From the study performed by Wu et al. (41) emerged that PAE compared with TURP resulted a cost-effectiveness strategy: the outcomes were comparable in terms of QALY (2.845 versus 2.854, respectively) but the PAE had lower costs respect to TURP ($2,934 vs $6,038). A comprehensive analysis (42) comparing several options in terms of clinical, quality of life and cost-effectiveness outcomes, generic combination therapy (CT), PUL, water vapor thermal therapy (WVTT), PVP and TURP, showed that water vapor thermal therapy could be the more convenient option over a short (1 year) and long time horizon (5 years). From the costs point of view, the least expensive treatment was WVTT whereas the most expensive was PUL ($2,019 versus $9,580, respectively).

NHS guidelines “UroLift for treating lower urinary tract symptoms of benign prostatic hyperplasia” highlighted that the UroLift System was likely to be cost saving respect the traditional approaches: over 5 years UroLift was estimated to save per person £981 respect to bipolar TURP, £1,242 compared with monopolar TURP and £1,230 respect to HoLEP. The cost savings were attributed to reduced length of stay and procedure time of UroLift respect to the other techniques (43).

At the present, we don’t not know studies evaluating the full spectrum of costs potentially associated with the treatment of LUTS, in particular exploring the dimensions of direct non-health costs and indirect costs and exploring a long-term horizon.

This translates into the lack of guidance for clinicians and healthcare providers, particularly for what concerns alternatives for PVP and TURP. As a consequence, TURP, despite implying higher costs and being limited to the inpatient setting, remains the preferred option and costs associated with LUTS and BPH still remain a challenge (16, 18).

Despite pharmacological treatment seems to hold the promise for lower costs in comparison with TURP, drug adherence, side-effects and lower degree of perception about symptoms improvements are all factors that limit its clinical and cost effectiveness (44, 45). Also, progression to invasive procedures should be taken into account. This is an important point, because TPLA might not only be an alternative to TURP or PVP (after pharmacological treatment fails), but also to pharmacological treatment itself, earlier in what is generally known as “patient journey”.

In the last years, several new MISTs emerged for the treatment of symptoms due to BPH aiming to improve the patient’s quality of life with similar efficacy compared to standard treatment. Clinical studies showed some degree of benefits associated with them although duration of follow up is still short to intermediate (16, 20–23). Moreover, many studies underline that the physician’s decision related to the treatment is also based on the patient’s preference.

TPLA minimally invasive technique is an emerging promising approach to be used in treatment of obstructive LUTS while preliminary results are promising both in terms of clinical and economic benefit. Also, as other MIST'S, the fact that the treatment could be delivered in an outpatient setting is of paramount importance not only to reduce costs, but also to avoid interruption or delay of care during a health emergency like eg the COVID-19 pandemic (24).

## Conclusion

Solid evidence about the economic implications about MIST'S and surgical approaches (like PVP or others) for the treatment of BPH are still lacking thus not providing guidance for clinically effective and sustainable choice among alternatives available for those patients. In particular, multicenter and long-term economic studies on all available treatment modalities are urgently needed to better assess the estimated disease burden. However, direct and indirect costs associated with EchoLaser TPLA are minimized versus TURP and laser vaporization/enucleation.

## Author contributions

VL and IP contributions include selecting studies, analyzing and presenting results. All authors contribute to write and revise the manuscript. All authors read and approved the final manuscript.

## Footnotes

1 The abstract with longest follow-up is referred to the study of de Rienzo et al. (23) where is reported 6 months follow-up.
