# Supplementary material for: Standard approach and future perspective for the management of benign prostatic hyperplasia from a health-economics point of view: the role of transperineal laser ablation
Source: Front Urol. 2023 Feb 24;3:1100386. doi: 10.3389/fruro.2023.1100386 (PMC12327305; doi:10.3389/fruro.2023.1100386)
Supplement: Supplementary file 1 [file Table_1.docx]

**Additional file 1.** Details of methods used to retrieve studies for the literature review

| **Search strategy** | |
| --- | --- |
| **PubMed** | (((“cost-benefit analysis”[MeSH Terms] OR (“cost-benefit”[All Fields] AND “analysis”[All Fields]) OR “cost-benefit analysis”[All Fields] OR (“cost”[All Fields] AND “effectiveness”[All Fields]) OR “cost effectiveness”[All Fields]) OR (“quality-adjusted life years”[MeSH Terms] OR (“quality-adjusted”[All Fields] AND “life”[All Fields] AND “years”[All Fields]) OR “quality-adjusted life years”[All Fields] OR “qaly”[All Fields]) OR ICER[All Fields] OR (direct[All Fields] AND (“health care costs”[MeSH Terms] OR (“health”[All Fields] AND “care”[All Fields] AND “costs”[All Fields]) OR “health care costs”[All Fields] OR (“healthcare”[All Fields] AND “cost”[All Fields]) OR “healthcare cost”[All Fields])) OR (direct[All Fields] AND non-healthcare[All Fields] AND (“economics”[Subheading] OR “economics”[All Fields] OR “cost”[All Fields] OR “costs and cost analysis”[MeSH Terms] OR (“costs”[All Fields] AND “cost”[All Fields] AND “analysis”[All Fields]) OR “costs and cost analysis”[All Fields])) OR (indirect[All Fields] AND (“economics”[Subheading] OR “economics”[All Fields] OR “cost”[All Fields] OR “costs and cost analysis”[MeSH Terms] OR (“costs”[All Fields] AND “cost”[All Fields] AND “analysis”[All Fields]) OR “costs and cost analysis”[All Fields]))) AND (“prostatic hyperplasia”[MeSH Terms] OR (“prostatic”[All Fields] AND “hyperplasia”[All Fields]) OR “prostatic hyperplasia”[All Fields] OR (“benign”[All Fields] AND “prostatic”[All Fields] AND “hyperplasia”[All Fields]) OR “benign prostatic hyperplasia”[All Fields])) AND English[Language]) AND (“2011”[PDAT] : “3000”[PDAT]) AND “humans”[MeSH Terms] |
| **Scopus** | TITLE-ABS ( “Cost effectiveness” OR “QALY” OR “ICER” OR “direct healthcare cost” OR “direct non-healthcare cost” OR “indirect cost” ) AND ( “Benign prostatic hyperplasia” ) AND ( LIMIT-TO ( PUBYEAR , 2021 ) OR ( PUBYEAR , 2020) OR ( PUBYEAR , 2019 ) ( PUBYEAR , 2018 ) OR LIMIT-TO ( PUBYEAR , 2017 ) OR LIMIT-TO ( PUBYEAR , 2016 ) OR LIMIT-TO ( PUBYEAR , 2015 ) OR LIMIT-TO ( PUBYEAR , 2014 ) OR LIMIT-TO ( PUBYEAR , 2013 ) OR LIMIT-TO ( PUBYEAR , 2012 ) OR LIMIT-TO ( PUBYEAR , 2011 ) ) AND ( LIMIT-TO ( DOCTYPE , “ar” ) OR LIMIT-TO ( DOCTYPE , “re” ) ) AND ( LIMIT-TO ( LANGUAGE , “English” ) ) AND ( LIMIT-TO ( SRCTYPE , “j” ) ) |
| **Inclusion and exclusion criteria** | |
| **Inclusion criteria** | Studies performed on humans, concerning adults (<=18 years), evaluating surgical approached for BPH/LUTS, considering the economic dimension of approaches and for which english full texts were available |
| **Exclusion criteria** | Literature review. Studies not considering the economic dimension |
